# Assembly of Nanoions via Electrostatic Interactions: Ion-Like Behavior of Charged Noble Metal Nanoclusters

**DOI:** 10.1038/srep03848

**Published:** 2014-01-24

**Authors:** Qiaofeng Yao, Zhentao Luo, Xun Yuan, Yue Yu, Chao Zhang, Jianping Xie, Jim Yang Lee

**Affiliations:** 1 Department of Chemical and Biomolecular Engineering, National University of Singapore, 10 Kent Ridge Crescent, Singapore 119260

## Abstract

The assembly of ultrasmall metal nanoclusters (NCs) is of interest to both basic and applied research as it facilitates the determination of cluster structures and the customization of cluster physicochemical properties. Here we present a facile and general approach to assemble noble metal NCs by selectively inducing electrostatic interactions between negatively-charged metal NCs and divalent cations. The charged metal NCs, which have well-defined sizes, charges and structures; and behave similarly to multivalent anions, can be considered as nanoions. These nanoions exhibit step-like assembly behavior when interacting with the counter cations – assembly only occurs when the solubility product (K_sp_) between the carboxylate ions on the NC surface and the divalent cations is exceeded. The assembly here is distinctively different from the random aggregation of colloidal particles by counter ions. The nanoions would assemble into fractal-like monodisperse spherical particles with a high order of regularity that mimic the assembly of ionic crystals.

Thiolate-protected noble metal (*e.g.*, Au and Ag) nanoclusters (NCs) are ultrasmall particles with a core size of 2 nm and lower^1^,^2^,^3^. They can be denoted as M_n_(SR)_m_ where n and m are respectively the numbers of metal atoms and thiolate ligands. Particles in this size range have strong quantum confinement effects^2^,^4^ and exhibit interesting molecular-like properties such as strong luminescence^5^,^6^,^7^,^8^,^9^,^10^,^11^,^12^ and enhanced catalytic activity^13^,^14^,^15^. Hence noble metal NCs could increase the diversity in functional materials for biomedical and catalytic applications. The physical and chemical properties of the NCs are dictated by their composition, size, and structure. Recent advances in NC chemistry have provided effective synthesis methods which can produce NCs with specific and tailorable attributes (*e.g.*, composition^16^,^17^,^18^,^19^, size^10^,^20^,^21^,^22^,^23^,^24^,^25^,^26^,^27^, and structure^28^,^29^).

Further customizations of cluster properties could be realized by constructing a higher order nanostructure via the assembly of discrete NCs. It is well-documented in the nanoparticle (NP, with core size > 3 nm) literature that close-packed assemblies of NPs may lead to enhanced and/or new collective properties^30^,^31^,^32^. Other than a means for property tuning, NC assembly also contributes to the total structure determination of NCs since the ordered assembly (crystallization) of NCs into a perfect single crystal enables the cluster structure analysis by X-ray crystallography^33^,^34^,^35^,^36^,^37^. Therefore, the development of effective and general approaches to assemble metal NCs is of interest to both basic and applied research.

The assembly of the relatively large metal NPs (>3 nm) is relatively well-established. A large number of NP assemblies with different diameters, geometries, and hierarchies have been demonstrated^38^,^39^,^40^,^41^,^42^,^43^,^44^,^45^,^46^,^47^. By comparison the assembly of ultrasmall metal NCs is a more recent development. Only a few Au NC species protected by aromatic thiolate/phosphine ligands have been assembled in organic solvents to date^33^,^34^,^35^,^36^,^37^,^48^. There are still no reports on the assembly of hydrophilic metal NCs in aqueous solution. The hydrophilic thiolate ligands on the NC surface do not contain the rigid aromatic rings which drive the assembly of Au NCs in organic solution through strong π-π interactions^9^,^25^,^49^. On the other hand the hydrophilic thiolate ligands on the NC surface may contain functionalities such as the carboxyl group (–COOH) which, upon deprotonation, can impart the NCs with a negative charge in aqueous solution^20^,^21^. Due to a very high ligand-to-metal ratio (~0.4 to 1 typically)^25^,^50^ and an ultrasmall size (<2 nm), a high charge density can be developed on the surface of the NCs. The high charge density and well-defined (atomically precise) size and structure (*e.g.*, spherical) qualify the hydrophilic metal NCs as a new family of ion mimics (“nanoions”) in the aqueous phase. We hypothesize that the ion-like features (high charge density and well-defined size and structure) could promote strong electrostatic interactions between the hydrophilic metal NCs and counter cations, in ways similar to the formation of ionic crystals from real ions.

Herein, we report a facile method to assemble negatively-charged metal NCs in water via the electrostatic interactions between the dissociated carboxyl groups on the NC surface and divalent counter cations (*e.g.*, Zn^2+^ and Cd^2+^) which are introduced to the solution. The divalent metal cations are used to electrostatically cross-link the negatively-charged NCs into large assemblies. An interesting ion-like behavior of the charged NCs was observed, where the assembly of NCs occurred in a step fashion and only when the metal-ion-to-thiolate-ligand ratio (denoted as R_[M]/[-SR]_) exceeded specific threshold values. The NC assemblies formed as such were spherical, fractal-like; and monodisperse in shape and size. The difference from the random aggregation of large colloidal NPs by counter ions is distinct and also suggests a high order of regularity in the assembly structure which is more akin to the formation of ionic crystals from real ions in solution. More interestingly, photoluminescence (PL) of the NCs was significantly enhanced after assembly, implying the existence of strong synergistic effects between the close-packed NC building blocks.

## Results

### Zn^2+^-induced assembly of Au NCs

**Figure 1:**
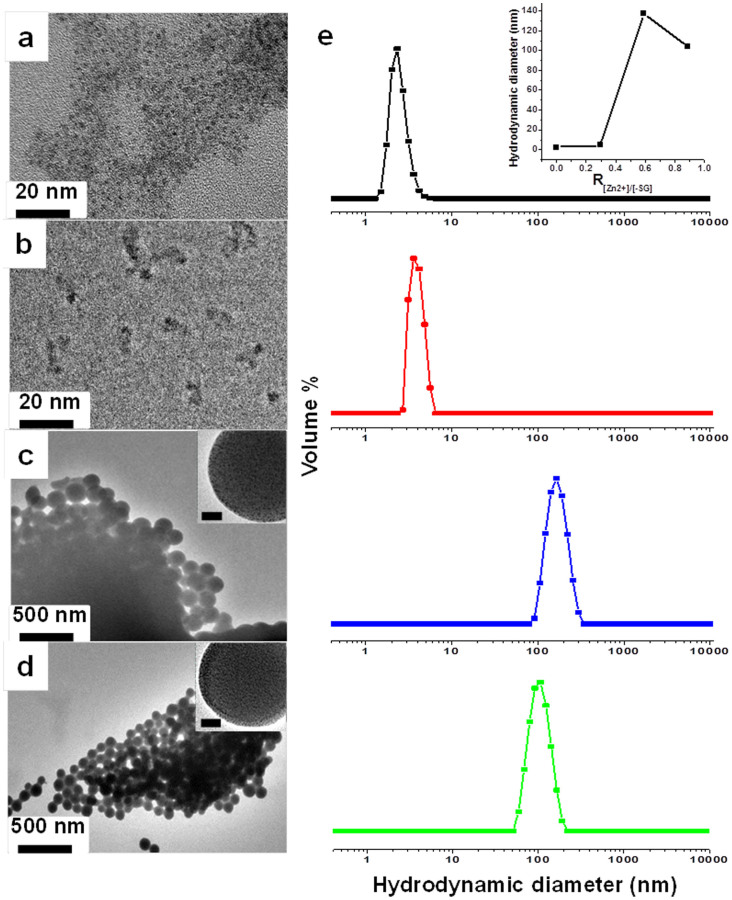
(a–d) TEM images of Au NCs assembled in different Zn^2+^concentrations: R_[Zn2+]/[-SG]_ = 0 (a), 0.3 (b), 0.6 (c), and 0.9 (d); the insets in (c) and (d) show the corresponding HR-TEM images (scale bar = 20 nm); (e) DLS analysis of Au NCs assembled at R_[Zn2+]/[-SG]_ = 0 (black), 0.3 (red), 0.6 (blue), and 0.9 (green); the inset shows the dependence of the hydrodynamic diameter of the assembly on R_[Zn2+]/[-SG]_.

As a proof-of-concept, highly luminescent Au NCs protected by the tripeptide glutathione (GSH or GS-H) were chosen as the model nanoion. The as-synthesized Au NCs showed strong orange emission which peaked at a wavelength of ~610 nm. As shown in Figure 1a, the orange-emitting Au NCs were small spheres with a core size below 2 nm. The Au NCs synthesized as such had a high GSH content on the NC surface (the GSH-to-Au-atom ratio was ~0.84 as reported in a previous study)^8^. The GSH ligands are likely to be present as oligomeric GS-[Au(I)-SG]_n_ motifs^8^ on the NC surface and determine the charge and surface properties of the Au NCs. Each GSH moiety carries two carboxyl groups with pK_a_ below 4^51^. Hence at the near neutral condition of pH 6.5 in our experiments, all of the carboxyl groups on the NC surface were deprotonated to give rise to a negative charge on the Au NCs. The ζ-potential plot in Supplementary Fig. S1 (see Supplementary Information) illustrates the pH dependence of the charge on the Au NCs.

Zn^2+^ was the counter cation used in this study. Zn^2+^ carries two positive charges and can electrostatically bind to two monovalent –COO^−^ anions. Therefore, Zn^2+^ can be used as a bifunctional cross-linker to electrostatically bridge between two negatively-charged Au NCs to build large NC assemblies. In a typical Zn^2+^-induced NC assembly, 1 mL of Au NCs at an optimized concentration of 0.46 mM [on Au atom basis as determined by inductively coupled plasma mass spectrometry (ICP-MS)] was mixed with 0.25 mL of ZnCl_2_ solution at a given concentration. The pH of the mixture was brought to ~6.5 by the addition of 0.1 M NaOH. The solution was well-mixed in a vortex mixer (1500 rpm, 30 s) and kept at room temperature for ~15 min to allow the reaction to complete. The procedure was repeated by using different concentrations of ZnCl_2_ while keeping the concentration of the Au NCs constant.

The size of the assembled NCs formed with different concentrations of Zn^2+^ was analyzed by transmission electron microscopy (TEM) and dynamic light scattering (DLS). The degree of assembly was determined by the ratio of divalent cations (Zn^2+^) to negatively-charged NCs (poly-anions). Since the latter depends on the GSH ligands on the NC surface, a controllable experimental variable would be the ratio of divalent cation (Zn^2+^) to GSH on the Au NC surface (taking into account that there are two carboxylate anions in one GSH moiety), termed as Zn^2+^-to-SG ratio or R_[Zn2+]/[-SG]_. If no Zn^2+^ ions were added to the Au NC solution [or R_[Zn2+]/[-SG]_ = 0], the Au NCs were well-dispersed in water and remained separated, as shown in the TEM image (Figure 1a). The discrete nature of the Au NCs was also supported by DLS measurements, where a hydrodynamic diameter (HD) of ~2.7 nm was calculated (Figure 1e, black line). When R_[Zn2+]/[-SG]_ was increased to 0.3, some small and irregularly-shaped NC assemblies began to appear in the TEM image (Figure 1b). The slight increase in HD in the DLS measurements (Figure 1e, red line) is consistent with the formation of small NC assemblies. The small amount of counter cations introduced to the NC solution at a low R_[Zn2+]/[-SG]_ value (0.3) could only induce controlled aggregation of the negatively-charged NCs to a limited extent; and only NC assemblies smaller than 10 nm were formed (Figure 1b). The small size of the NC assemblies was also corroborated by UV-vis spectroscopy (Supplementary Fig. S2) where scattering intensity increase (indication of a significant particle size increase)^8^ was not found relative to the discrete Au NCs.

Fractal-like spheres displaying limited self-similarity^52^ (spherical nanoion to spherical colloidal assembly at the scale of ~1 and 100 nm, respectively) were present in the TEM image when R_[Zn2+]/[-SG]_ was increased to 0.6 (Figure 1c). The spherical assemblies had a uniform core size of 136.2 nm (100 colloidal spheres counted) and a HD of ~137.6 nm (Figure 1e, blue line). The assembled structure at a higher R_[Zn2+]/[-SG]_ value of 0.9 was visually similar (Figure 1d). The diameter of the NC assemblies was however smaller, at ~92.8 nm (100 colloidal spheres counted) and likewise the measured HD decreased to ~93.5 nm (Figure 1e, green line). An increase in the scattering intensity of the NC assemblies was noted when R_[Zn2+]/[-SG]_ was increased from 0.6 to 0.9 (Supplementary Fig. S2). This is an indication of the increase in the number of NC assemblies at R_[Zn2+]/[-SG]_ of 0.9. Hence at the higher R_[Zn2+]/[-SG]_ value of 0.9, there were more NC assemblies formed but the size was smaller (*vs* R_[Zn2+]/[-SG]_ = 0.6). This finding can be understood based on the common nucleation-growth mechanism^53^. At a higher R_[Zn2+]/[-SG]_ (0.9), more Zn^2+^ ions were available as electrostatic cross-linkers, leading to a faster formation of “assembly nuclei”. More “nucleation sites” were available for growth through continuous assembly of NCs. Consequently smaller NC assemblies were formed when the same NC concentration was used. The dependence of the NC assembly size on the concentration of the counter cations (Zn^2+^) is summarized in the inset of Figure 1e, where the maximum in the size of the NC assemblies was found to be around ~137.6 nm.

**Figure 2:**
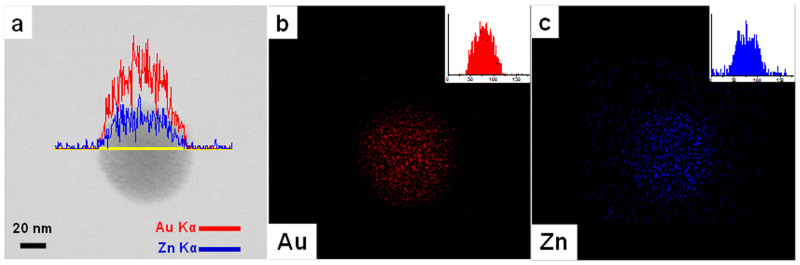
(a) Scanning transmission electron microscopy (STEM) image of a representative Au NC colloidal sphere assembled at R_[Zn2+]/[-SG]_ = 0.6; (b–c) EDX elemental maps of the circular region in (a) in terms of Au (b), and Zn (c); the insets in (b) and (c) show respectively the Au and Zn signals in the EDX line scan across the yellow line in (a).

The structure and composition of fractal-like NC assemblies assimilated by Zn^2+^ were also examined by high-resolution TEM (HR-TEM). The HR-TEM images of assemblies formed at R_[Zn2+]/[-SG]_ of 0.6 and 0.9 are shown as insets in Figure 1c and 1d respectively, where NCs smaller than 2 nm are clearly visible within the assembly. UV-vis spectroscopy also provided supporting evidence for the assimilation of primary NCs (Supplementary Fig. S2). The characteristic absorption peak of isolated (primary) Au NCs at ~400 nm was mostly retained in the UV-vis spectra of the NC assemblies. The surface plasmon resonance (SPR) of large spherical Au NPs, which absorbs at ~520 nm (for ~50 nm Au NPs)^54^ was conspicuously absent, suggesting that the size and structure of the Au NC building blocks were preserved in the assemblies. No increase in the size of the NC building blocks was detected in the assemblies, confirming that the ion-induced NC assembly is a soft approach. The relatively weak electrostatic interaction between the NCs and the counter ions was unable to alter the size and structure of the NC building blocks. The chemical composition of the NC assemblies was analyzed by energy dispersion X-ray spectroscopy (EDX). Line scan analysis across the diameter of a representative assembly (Figure 2a) and the elemental maps of the latter confirm the uniform distribution of Au (Figure 2b) and Zn (Figure 2c) throughout the colloidal sphere, suggesting that the spherical assembly was formed by the assimilation of Au NCs by Zn^2+^ ions.

### Ion-like behavior of Au NCs

The assembly of Au NCs exhibited an intriguing ion-like behavior, where a step-wise dependence on the concentration of counter cations (Zn^2+^) was observed, as shown in the insets of Figure 1e and Supplementary Fig. S2. The negatively-charged NCs were initially inert to the addition of a small amount of Zn^2+^ up to a threshold value of R_[Zn2+]/[-SG]_ (~0.3). Increase in the R_[Zn2+]/[-SG]_ value beyond this threshold value (*e.g.*, 0.3 < R_[Zn2+]/[-SG]_ < 0.6) triggered the formation of spherical NC assemblies, similar to the formation of precipitates beyond the solubility limit defined by the solubility product K_sp_. In precipitation, taking A^+^ + B^−^ = AB↓ as an example, the ionic product Q = [A^+^][B^−^] is always a constant (K_sp_) in a solution saturated with the precipitate AB. Similarly, a constant Q value ( = [COO^−^]^2^[Zn^2+^] considering the formation of –COO^−^**···**Zn^2+^**···**^−^OOC– ion pairs) was also observed in the “saturated solutions” of negatively-charged NCs and counter cations (Zn^2+^). The Q values (determined by ICP-MS) obtained in R_[Zn2+]/[-SG]_ of 0.6 and 0.9 were 3.31 × 10^−11^ and 3.35 × 10^−11^ M^3^ respectively. The K_sp_ was therefore estimated to be 3.33 × 10^−11^ M^3^. The high ligand-to-metal ratio (the SG-to-Au ratio is ~0.84) and the uniform size and structure of the Au NCs are key contributions to their ion-like properties (step-like assembly). The possession of ion-like structural features in the Au NCs not only made it possible for the step-like assembly, but also produced an ordered structure mimicking the formation of ionic crystalline compounds. In nature oppositely charged ions are often packaged into crystalline ionic compounds with long-range order. Similarly, the NC assemblies here adopted a fractal-like spherical geometry (Figure 1c and 1d). It should be reiterated that such fractal-like assembly was different from the counter-ion-induced assembly of large NPs (>3 nm) where the aggregates display only a low symmetry^55^,^56^. However, it should be noted that the spherical NC assemblies did not exhibit long-range crystalline order. This could be attributed to the relatively large difference in size between the negatively-charged NCs and the counter cations (Zn^2+^).

**Figure 3:**
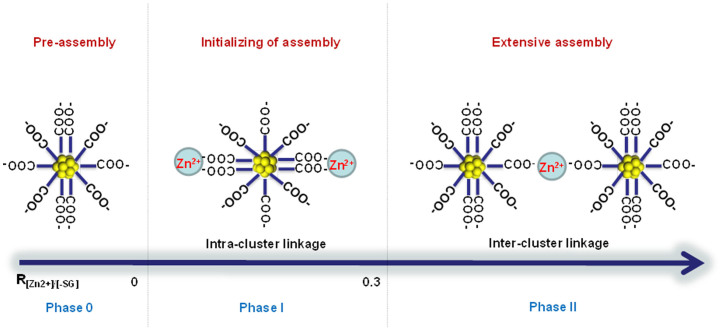
Proposed mechanism for Zn^2+^-induced assembly of Au NCs.

The counter cations Zn^2+^ exerted their influence on the assembly of negatively-charged Au NCs through principally two effects. The first was the screening effect where Zn^2+^ reduced the negative charge on the Au NC surface through the formation of *intra*-cluster –COO^−^**···**Zn^2+^**···**^−^OOC– electrostatic linkage (referred to henceforth as *intra*-EL). The second was the bridging effect forming *inter*-cluster –COO^−^**···**Zn^2+^**···**^−^OOC– electrostatic linkages (*inter*-ELs). The bridging effect brought two separate NCs together while *intra*-EL did not bring about NC assembly *per se*. These two effects are illustrated schematically in Figure 3. At low R_[Zn2+]/[-SG]_ (~0.3), the amount of Zn^2+^ in the NC solution was small and Zn^2+^ tended to bind to carboxylate anions on the same NC, thus leading to the formation of *intra*-ELs. This was the phase I stage in the assembly of NCs. Charge screening effect was dominant in this stage, as evidenced by the rapid increase in the ζ-potential of the NCs with the increase in the Zn^2+^ concentration (R_[Zn2+]/[-SG]_ from 0 to 0.3, Supplementary Fig. S3). The formation of *intra*-ELs gradually approached saturation with the continuing increase in Zn^2+^ concentration. At the threshold value of R_[Zn2+]/[-SG]_ (determined to be ~0.3 in this study), *inter*-ELs began to form resulting in the formation of small and irregularly-shaped NC assemblies due to partial bridging effect (Figure 1b). Further increase in the Zn^2+^ concentration increased the extent of *inter*-ELs (ionic cross-linking) until the step-like assembly of the Au NCs occurred, the inception of which is identified as phase II in Figure 3. For example, at R_[Zn2+]/[-SG]_ = 0.6, the Au NCs were extensively assimilated into colloidal spheres (Figure 1c). However, the ζ-potential of the NCs (Supplementary Fig. S3) was nearly unchanged when R_[Zn2+]/[-SG]_ was increased from 0.3 to 0.6. This observation suggests that in the concentration range of 0.3 < R_[Zn2+]/[-SG]_ < 0.6, the charge screening of Zn^2+^ was nearly constant and the bridging effect of Zn^2+^ was more important than the screening effect. A further increase in the Zn^2+^ concentration from R_[Zn2+]/[-SG]_ = 0.6 to 0.9 led to the gradual depletion of Au NCs in the solution, and the completion of the NC assembly process. The gradual depletion of NCs was accompanied by the accumulation of excess Zn^2+^ through adsorption on the surface of NC assemblies, which resulted in a second series of ζ-potential increase of the NC solution (Supplementary Fig. S3).

**Figure 4:**
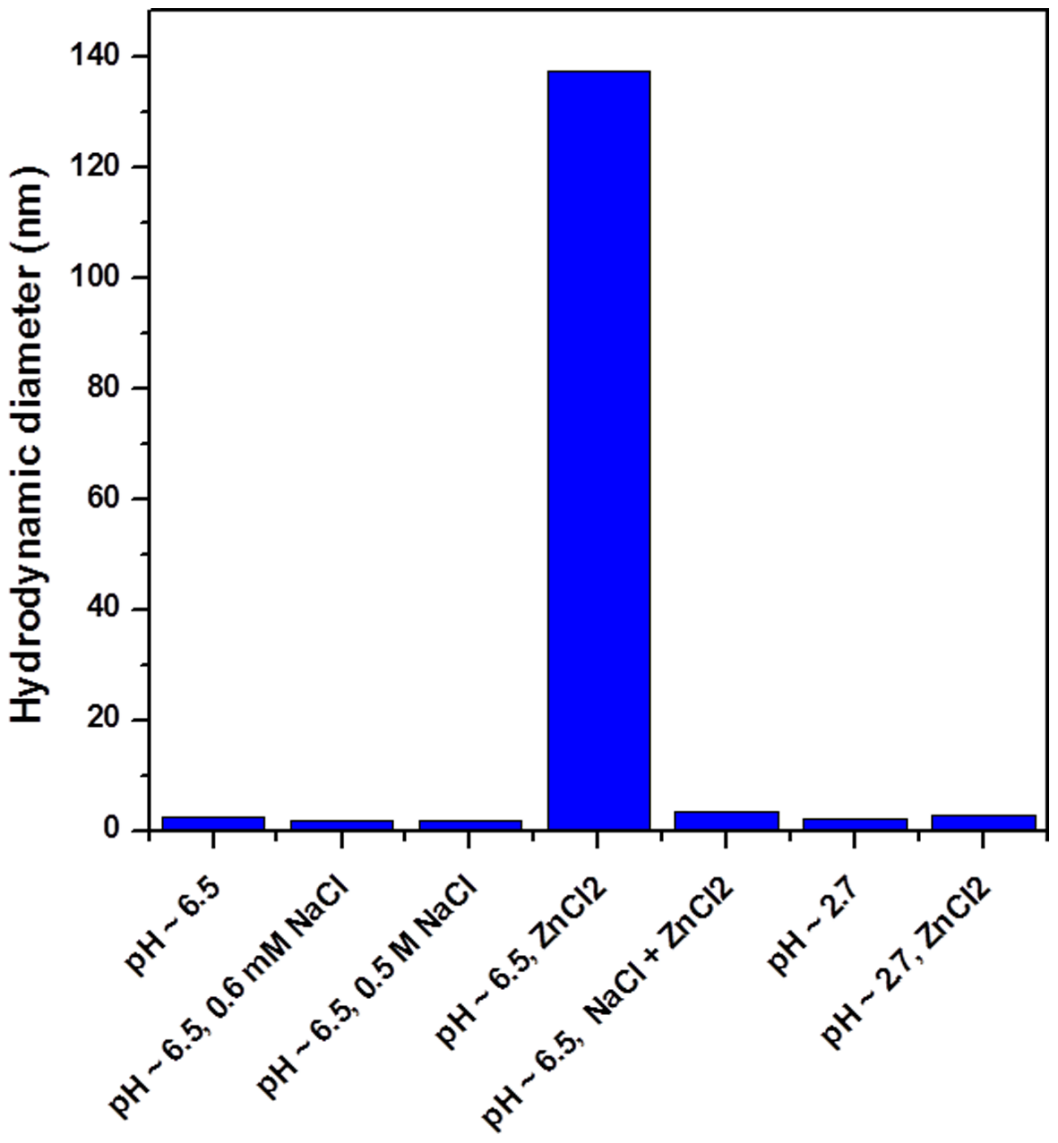
Measured hydrodynamic diameters of assembled/disassembled Au NCs at different pH and in the presence of different ions. The assembly with Zn^2+^ was carried out at R_[Zn2+]/[-SG]_ = 0.6.

Several independent measurements also suggest the bridging effect of divalent Zn^2+^ ions as the primary factor in the assembly of Au NCs. First, common monovalent cations such as Na^+^ ions (from dissolved NaCl), which could also screen the surface charge of Au NCs, were unable to cause a similar assembly of the Au NCs. Au NCs remained separate in 0.6 mM NaCl solution (same ionic strength as the 0.2 mM ZnCl_2_ used to provide R_[Zn2+]/[-SG]_ = 0.6, please refer to the Methods section for the details of the ionic strength calculation). The HD measurement in Figure 4 (second column) indicated likewise. A further increase in the ionic strength of the NaCl solution (*e.g.*, by increasing the NaCl concentration to 0.5 M) also did not help to bring about the assembly of Au NCs (Figure 4, third column). Hence the screening effect of cations on the surface charge of the NCs is not critical to the assembly of NCs. The increase in the solution ionic strength could however disrupt the electrostatic interaction between Zn^2+^ and carboxylate anions. The presence of NaCl in large excess in the Au NC solution (~0.5 M in the final reaction mixture) prior to ZnCl_2_ addition could suppress the assembly to a great extent. The HD of the NCs in the presence of both Na^+^ and Zn^2+^ was also the same as that of discrete NCs, thereby suggesting no significant assembly of the Au NCs (Figure 4, fifth column).

**Figure 5:**
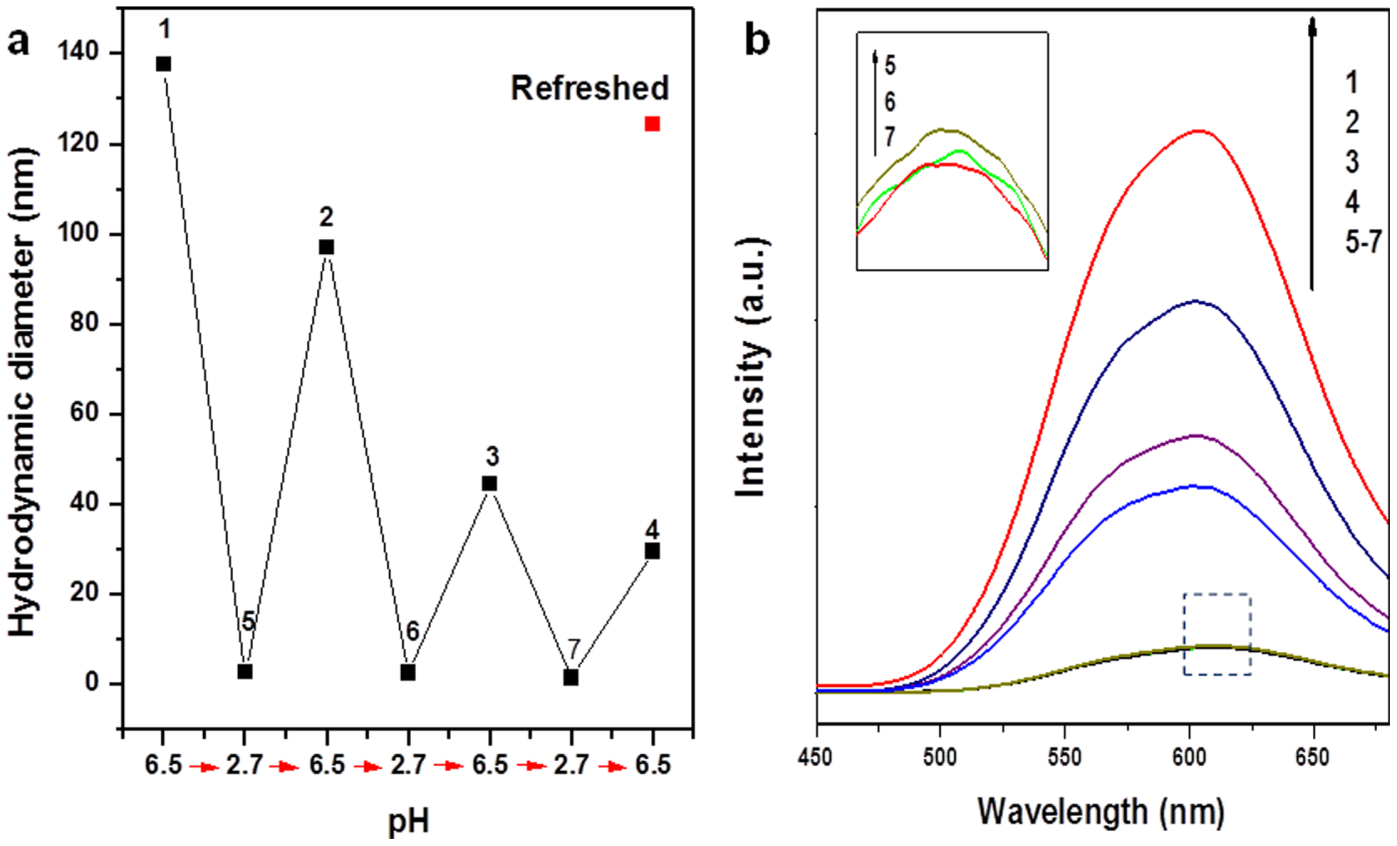
Effects of pH cycling on the hydrodynamic diameters (a) and photoemission spectra (b, excited at 365 nm) of Au NCs assembled by Zn^2+^. The Au NCs were initially assembled at pH ~6.5 (**#1**), and then pH was varied in the sequence of ~2.7 (**#5**) -- 6.5 (**#2**) -- 2.7 (**#6**) -- 6.5 (**#3**) -- 2.7 (**#7**) -- 6.5 (**#4**). In a separate experiment, solution **#7** was dialyzed overnight followed by reassembly with Zn^2+^ at pH 6.5. The hydrodynamic diameter of the reassembled “refreshed” Au NCs was recorded as the red dot in (a). The inset in (b) shows the expanded view of the circled area. All assemblies were carried out at R_[Zn2+]/[-SG]_ = 0.6.

Excess H^+^ had the same effect as Na^+^ in disrupting the assembly of NCs by Zn^2+^. For example, by adjusting the pH of the NC solution to ~2.7 (a value lower than the isoelectric point of Au NCs, Supplementary Fig. S1), the assembly of Au NCs was significantly inhibited, as shown by the nearly invariant HD of the NCs in the presence and absence of Zn^2+^ at pH ~2.7 (Figure 4, the last two columns). More interestingly, the assembly and disassembly of the Au NCs could be reversed by cycling the pH of the solution between 6.5 and 2.7. The zigzag changes in the HD of the Au NCs under pH cycling (Figure 5a) indicate that the Au NCs could undergo repeated assembly and disassembly at pH 6.5 and 2.7 respectively.

There was irreversibility shown, however, as the HD of the Au NCs at pH 6.5 gradually decreased with the increase in the cycle number. This trend could be attributed to the slow accumulation of ionic strength due to the way pH cycling was implemented: The pH of the solution was adjusted by the addition of a given amount of 0.1 M HCl or 0.1 M NaOH, leading to the gradual buildup of ionic strength with the increase in the cycle number. A higher ionic strength of the solution increased the screening effect on the electrostatic interactions between Zn^2+^ and negatively-charged NCs, thus leading to a smaller degree of assembly (decreasing size of the NC assemblies). The ionic-strength-induced irreversibility could be eliminated by removing the excess small ions. We “refreshed” the Au NC solution at the lower pH of cycle 3 (corresponding to point 7 in Figure 5a) by dialysis, where a semipermeable membrane with a molecular weight cut off (MWCO) of 3500 Da was used to filter away the small ions (*e.g.*, H^+^, Na^+^, Zn^2+^ and Cl^−^). After pH adjustment to 6.5 and Zn^2+^ addition, a HD of 124.4 nm (the red dot in Figure 5a) could be obtained from the “refreshed” Au NC solution, similar to the HD assembled at pH = 6.5 in cycle 1 from a fresh Au NC solution (137.6 nm, point 1 in Figure 5a). This observation led us to conclude that the reversibility of assembly and disassembly could be greatly improved through the control of the ionic strength.

### Assembly induced photoluminescent enhancement

**Figure 6:**
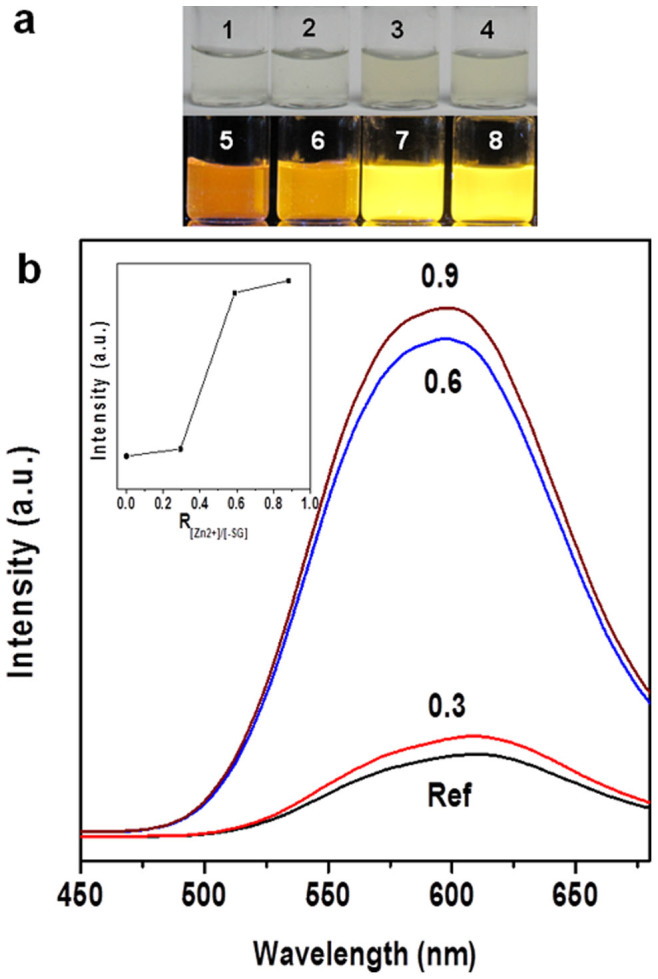
(a) Digital photos of Au NCs assembled at various R_[Zn2+]/[-SG]_ values: 0 (**#1** and **#5**), 0.3 (**#2** and **#6**), 0.6 (**#3** and **#7**), and 0.9 (**#4** and **#8**); #1 ~ 4 were taken under normal light while #5 ~ 8 were taken under UV illumination (365 nm); (b) Photoemission spectra (exited at 365 nm) of Au NCs assembled under different R_[Zn2+]/[-SG]_ values: 0 (black), 0.3 (red), 0.6 (blue), and 0.9 (purple); the inset shows the emission intensity maximum as a function of R_[Zn2+]/[-SG]_.

The physical and chemical properties of the Au NCs could be altered by proximity effects in an ionically cross-linked close-packed structure. One most interesting property of the Au NCs is their strong optical luminescence, as shown in Figure 6 (black line). The as-synthesized Au NCs had a unique Au(0)@Au(I)-thiolate core-shell structure, where the *intra*-cluster aurophilic interaction [Au(I)-Au(I)] contributed to the PL of the NCs^8^,^57^,^58^. The distance between the Au NCs could be significantly shortened after assembly to promote *inter*-cluster aurophilic interaction. The increase in *inter*-cluster aurophilic interaction could increase the PL of the NCs. The assembly of Au NCs could also reduce non-irradiative relaxation of excited electrons, which also enhances the PL of the NCs^59^,^60^. For example, the bridging Zn^2+^ between neighboring carboxylate anions (via either an *intra*- or *inter*-EL) could inhibit the vibration or rotation of the thiolate ligands, which is one of the non-irradiative relaxation pathways^8^. Experimentally the PL (at 610 nm) of the NC assemblies (Figure 6) was significantly higher than that of discrete Au NCs. In order to rule out the possibility of PL enhancement through the formation of Zn^2+^-thiolate (-SG) complexes, a control experiment was carried out by mixing Zn^2+^ and GSH in water. The resultant Zn-SG complexes did not exhibit any luminescence (data not shown). Two other experimental observations also provided supporting evidence on proximity-induced properties in the NC assemblies. First, as shown in the inset of Figure 6b, the PL enhancement effect also displayed a similar step-like trend with the increase of the Zn^2+^ concentration, which corresponds well with the step-like assembly of the Au NCs. Second, the PL enhancement could also be turned on or off corresponding to the assembly or disassembly of Au NCs in pH cycling (Figure 5b).

## Discussion

The ion-induced assembly of NCs developed in this study is expected to be generic, and hence should be applicable to other charged metal NCs and divalent counter ions. For example, other divalent cations, such as Cd^2+^, may be used to substitute for Zn^2+^. Similar spherical NC assemblies were indeed obtained by using Cd^2+^ as the counter cations (Supplementary Fig. S4). Similarly, the PL intensity of the NCs assimilated by Cd^2+^ was also significantly higher than that of discrete Au NCs (Supplementary Fig. S4). The ion-induced assembly of NCs could also be extended to other nanoions; *e.g.*, red-emitting Ag NCs protected by GSH. As shown in Supplementary Fig. S5, the addition of Zn^2+^ to the Ag NC solution also induced the assembly of Ag NCs, leading to the formation of spherical NC assemblies. Enhanced PL was also detected for the Ag NC assemblies (Supplementary Fig. S5).

In summary, we have developed a facile and general method to assemble charged noble metal NCs in water based on the electrostatic interaction between oppositely-charged entities. Negatively-charged Au NCs were assembled into monodisperse and fractal-like spherical assemblies by divalent counter cations such as Zn^2+^ and Cd^2+^, via the formation of *inter*-cluster electrostatic linkages (*inter*-EL) of the type –COO^−^**···**M^2+^**···**^−^OOC–. Owing to their high charge density, ultrasmall size, and structural uniformity, the Au NCs may be regarded as an ion-mimic nanomaterial, exhibiting ion-like properties of step-like assembly dictated by K_sp_ and a high structural order in the assembly. The bridging effect of the divalent cations is essential to the formation of NC assemblies. A strong synergy between the NCs due to the close-packed order in the assembly resulting in the PL enhancement of NCs was also demonstrated in this study. The NC assemblies and the assembly methods in this study are of interest not only because they provide a simple and general method to generate a superstructure of functional noble metal NCs, which is of interest to both basic and applied research; but also because they exemplify a NC surface with charged hydrophilic ligands as three-dimensional nanoions, with the potential to mimic some of the useful behavior of real ions in various practical settings.

## Methods

### Materials

Hydrogen tetrachloroaurate(III) trihydrate (HAuCl_4_·3H_2_O), L-glutathione reduced form (GSH), and sodium borohydride (NaBH_4_) from Sigma Aldrich; hydrochloric acid (HCl) from J. T. Baker; zinc chloride (ZnCl_2_), silver nitrate (AgNO_3_) and sodium hydroxide (NaOH) from Merck; and cadmium chloride (CdCl_2_) from Strem were used as received without further purification. Ultrapure Milipore water (18.2 MΩ) was used in the preparation of all aqueous solutions. All glassware was washed with *aqua regia* and rinsed with ethanol and ultrapure water before use.

### Synthesis of orange-emitting Au NCs

A more detailed description of the synthesis method can be found in the original work^8^. Briefly, 0.5 mL of 20 mM HAuCl_4_ and 0.20 mL of 100 mM GSH aqueous solutions were mixed with 4.35 mL of ultrapure water at room temperature (25°C). After incubation under gentle stirring (500 rpm) at 70°C for 24 h, bright orange-emitting Au NCs were formed.

### Synthesis of red-emitting Ag NCs

Red-emitting Ag NCs were prepared by a cyclic reduction-decomposition method detailed elsewhere^10^. Briefly, 125 μL of 20 mM AgNO_3_ and 150 μL of 50 mM GSH aqueous solutions were added to 4.85 mL of ultrapure water under vigorous stirring (~1200 rpm) to form Ag(I)-thiolate complexes. 50 μL of 112 mM NaBH_4_ aqueous solution was then introduced to reduce the Ag(I)-thiolate complexes, giving rise to a deep-red solution of Ag NCs within 5 min. The As-formed Ag NC solution (deep-red) was allowed to decompose to Ag(I)-thiolate complexes (colorless) after ~3 h of incubation at room temperature (25°C). 50 μL of 112 mM NaBH_4_ aqueous solution was then added to this colorless solution under vigorous stirring (~1200 rpm). The color of solution turned from colorless to light-brown within 15 min. This light-brown Ag NC solution was allowed to stand (without stirring) at room temperature (25°C) for 8 h to form red-emitting Ag NCs in aqueous solution.

### Ion-induced assembly of orange-emitting Au NCs

ZnCl_2_ and CdCl_2_ aqueous solutions in specific concentrations were freshly prepared by dissolving calculated amounts of ZnCl_2_ and CdCl_2_ in ultrapure water respectively. The pH of each metal salt solution was adjusted to ~6.5 by adding 0.1 M NaOH. The As-prepared Au NC aqueous solution was diluted 4 times (4× diluted) and the pH was adjusted to ~6.5 before use. In a typical assembly (taking R_[Zn2+]/[-SG]_ = 0.6 as an example), 0.25 mL of 1 mM ZnCl_2_ was quickly added to 1 mL of the diluted Au NC solution, followed by mixing on a vortex mixer (1500 rpm for 30 s). The pH of the mixture was maintained at ~6.5. Subsequently, the mixture was kept still at room temperature (25°C) for 15 min to allow the assembly to complete. Assembly of Au NCs by Zn^2+^ in other concentrations and by Cd^2+^ solution were also carried out likewise, using different metal salt solutions before mixing.

### Ion-induced assembly of red-emitting Ag NCs

The pH of Ag NC as well as 2 mM ZnCl_2_ aqueous solution was brought to ~6.5 before use. Similar to the Zn^2+^ induced assembly of Au NCs, 1 mL of Ag NC and 0.25 mL of 2 mM ZnCl_2_ aqueous solutions were mixed while pH was maintained at ~6.5, followed by still incubation at room temperature (25°C) for 15 min.

### Dialysis of NC solution

5 mL of NC solution was sealed in a dialysis bag made of a semipermeable membrane (MWCO = 3500 Da, Spectra/Por®), followed by immersion into 5 L of ultrapure water. The dialysis process lasted overnight under moderate stirring (1000 rpm).

### Estimation of the ionic strength of ZnCl_2_ and NaCl in the solutions

The ionic strength of NaCl or ZnCl_2_ solution was calculated by the equation, I = 0.5 ∑ c_i_z_i_^2^, where I is the ionic strength, c_i_ is the concentration of a specific ion, and z_i_ is the specific charge number of the ion.

### Characterization

pH was monitored by a Mettler Toledo FE 20 pH-meter. UV-vis absorption and photoluminescence (PL) spectra were recorded by a Shimadzu UV-1800 spectrometer and a PerkinElmer LS-55 fluorescence spectrometer respectively. Transmission electron microscopy (TEM), scanning transmission electron microscopy (STEM), and Energy-dispersive X-ray spectroscopy (EDX) elemental analysis were performed on a JEOL JEM 2010 microscope operating at 200 kV. The size and ζ-potential of NCs before and after assembly were measured by dynamic light scattering (DLS) and electrophoresis light scattering (ELS) on a Malvern Zetasizer Nano ZS, respectively. The samples were centrifuged by an Eppendorf Centrifuge 5424. The Au and Zn contents in the samples were analyzed by inductively coupled plasma mass spectrometry (ICP-MS) measurement on an Agilent 7500A. The GSH and carboxyl group contents were calculated based on the Au content via the relation Au:GSH:COOH = 1:0.84:1.68^8^.

## Author Contributions

This project was carried out under the supervision of J.Y.L. and J.X. J.Y.L., J.X. and Q.Y. designed the experiments. Q.Y., Z.L. and X.Y. performed the experiments and Q.Y., Y.Y. and C.Z. analyzed the results. All authors participated in the discussion of results and manuscript preparation.

## Supplementary Material

Supplementary Information Supplementary Information
