# Supplementary material for: Assembly of Nanoions via Electrostatic Interactions: Ion-Like Behavior of Charged Noble Metal Nanoclusters
Source: Sci Rep. 2014 Jan 24;4:3848. doi: 10.1038/srep03848 (PMC3900922; doi:10.1038/srep03848)
Supplement: Supplementary Information [file srep03848-s1.pdf]

Supplementary Information for

Assembly of Nanoions via Electrostatic Interactions: Ion-Like Behavior of Charged Noble Metal Nanoclusters

Qiaofeng Yao, Zhentao Luo, Xun Yuan, Yue Yu, Chao Zhang, Jianping Xie* and Jim Yang Lee*

Department of Chemical and Biomolecular Engineering, National University of Singapore,

10 Kent Ridge Crescent, Singapore 119260

*To whom correspondence should be addressed. Emails: cheleejy@nus.edu.sg (JYL) / chexiej@nus.edu.sg (JX)

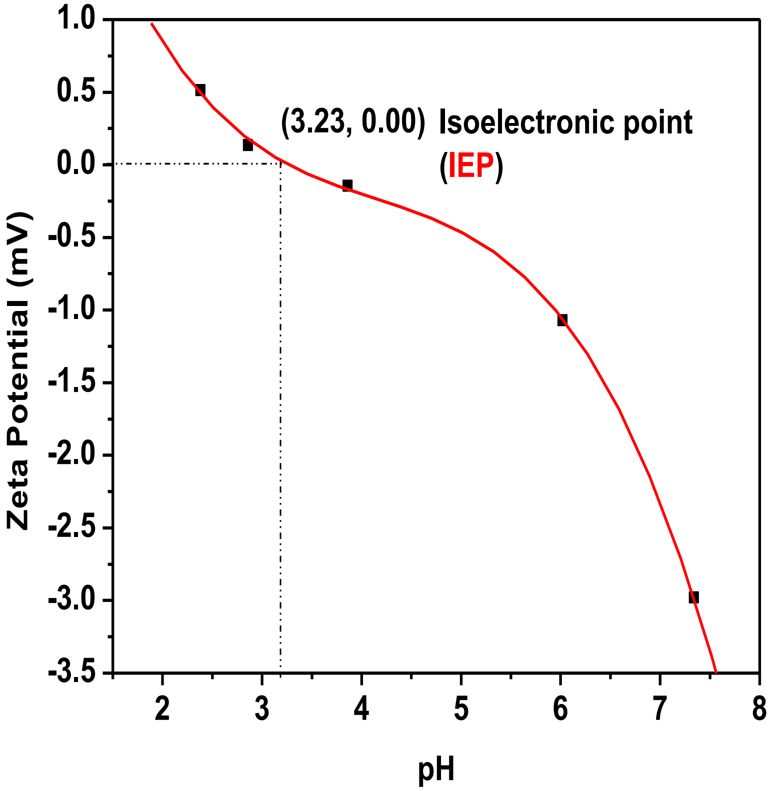

Figure S1 pH dependence of the ζ-potential of orange-emitting Au NCs. The isoelectric point (IEP) of the Au NCs was estimated to be 3.23.

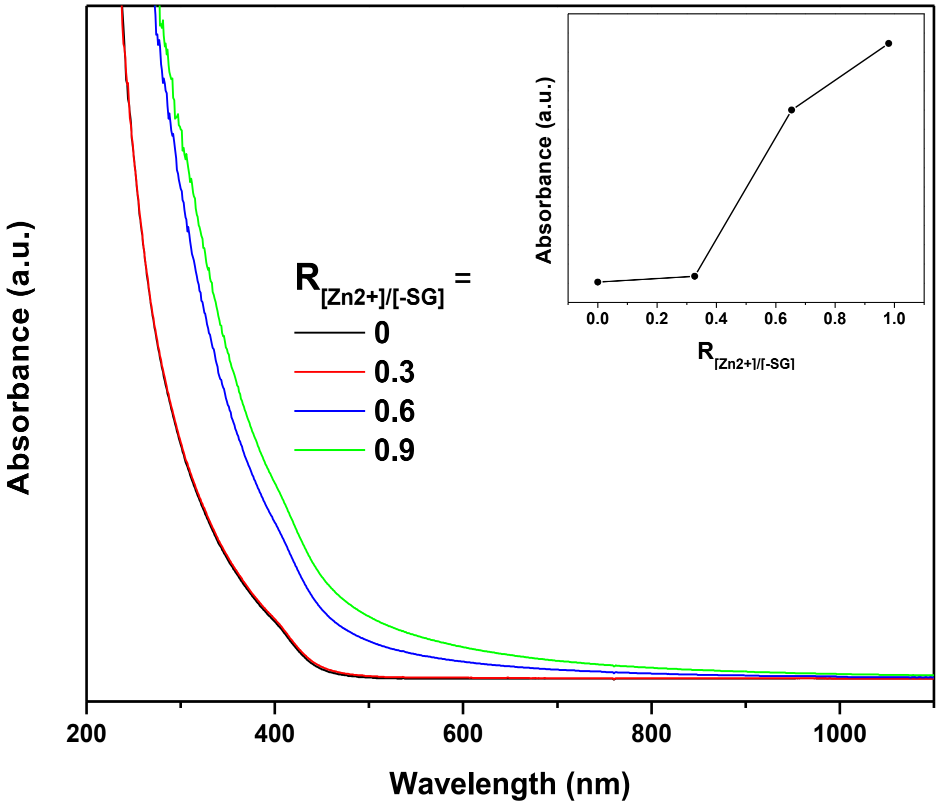

Figure S2 UV-vis absorption spectra of Au NCs assembled at different $R_{[Zn2+]/[-SG]}$ values; the inset shows the absorbance at 400 nm as a function of $R_{[Zn2+]/[-SG]}$.

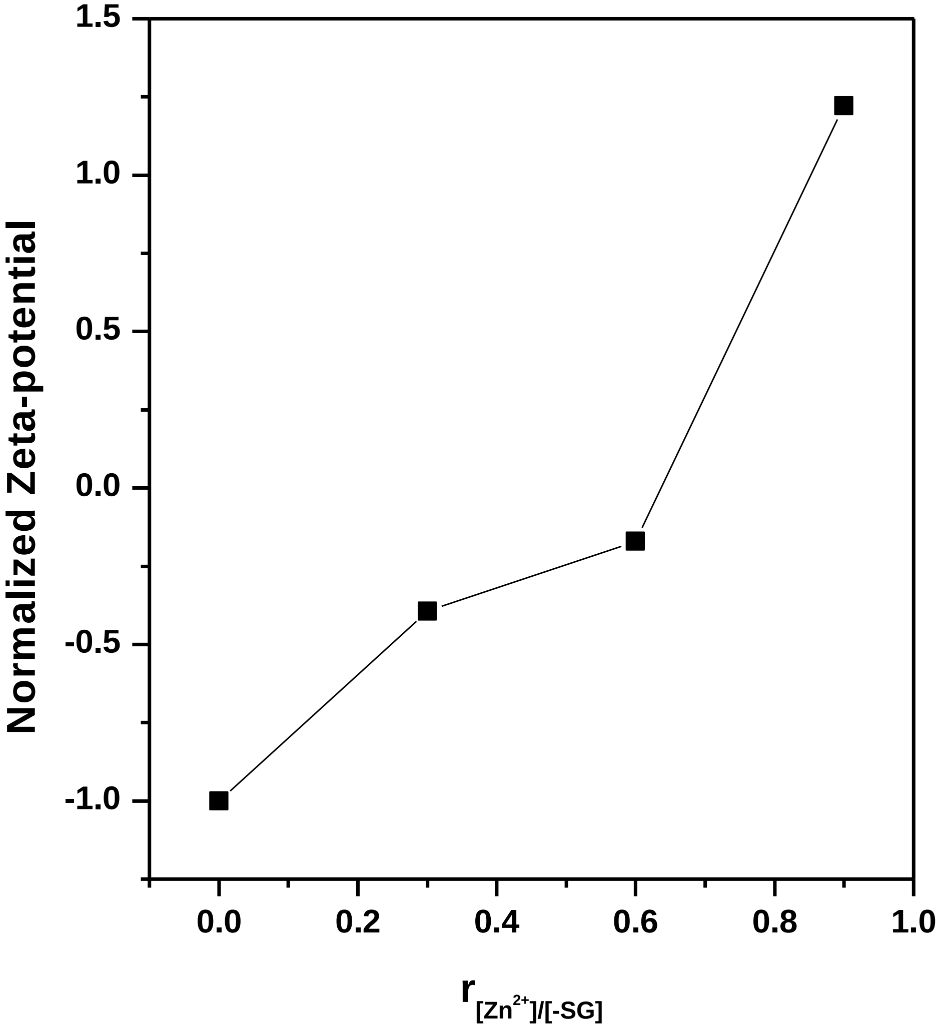

Figure S3 ζ-potential of Au NCs assembled under different $R_{[Zn2+]/[-SG]}$ values. The ζ-potential at $R_{[Zn2+]/[-SG]} = 0$ was arbitrarily set at -1 for ease of comparison.

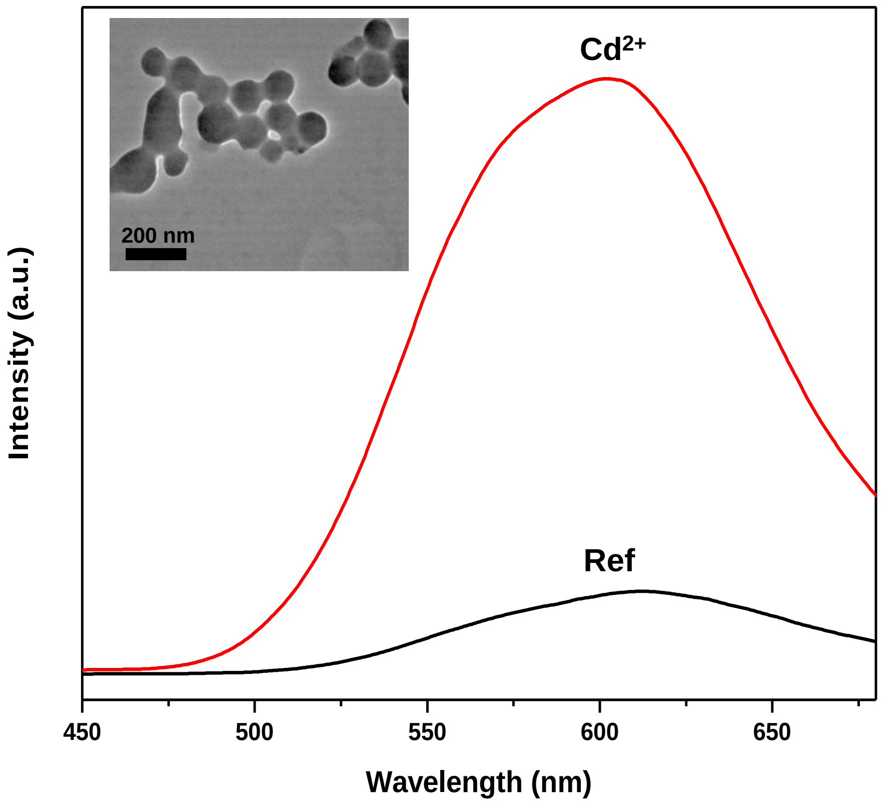

Figure S4 Photoemission spectrum (after excitation at 365 nm) of Au NCs assembled by Cd^{2+} (red lines) with corresponding TEM image shown as an inset. The black line corresponds to Au NCs without Cd^{2+}.

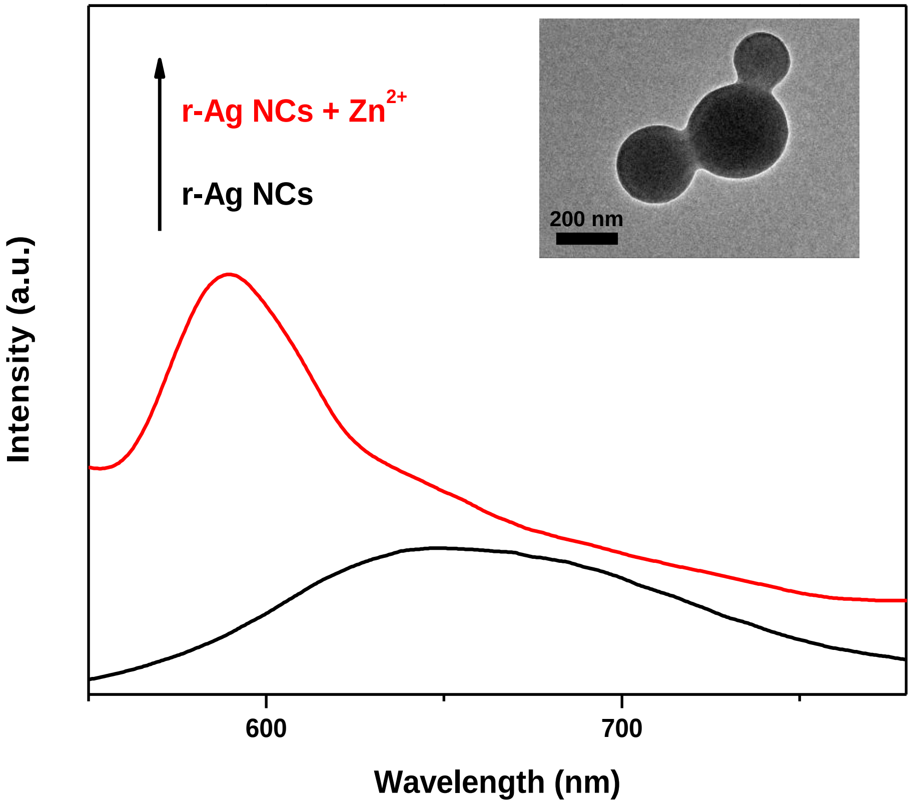

Figure S5 Photoemission spectrum (after excitation at 440 nm) of red-emitting Ag NCs assembled by Zn^{2+} (red lines) with the corresponding TEM image shown as an inset. The black line corresponds to Ag NCs without Zn^{2+}.
